# Supplementary figures and images for: Relying on known or exploring for new? Movement patterns and reproductive resource use in a tadpole-transporting frog
Source: PeerJ. 2017 Aug 29;5:e3745. doi: 10.7717/peerj.3745 (PMC5580388; doi:10.7717/peerj.3745)

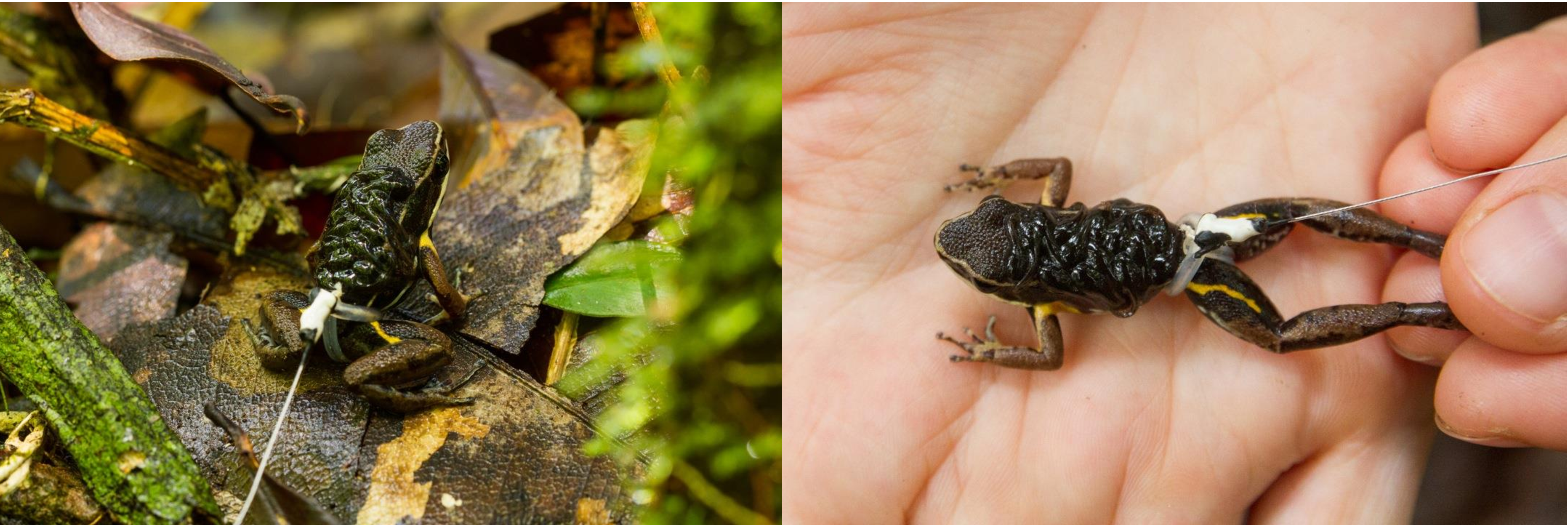

Supplement: Figure S1 — The tag consists of a silicon tube around the waist, with an additional silicone strap between the hind legs, a small diode (beneath the white sealing) and a dipole antenna made of flexible coated wire. [file peerj-05-3745-s002.png]

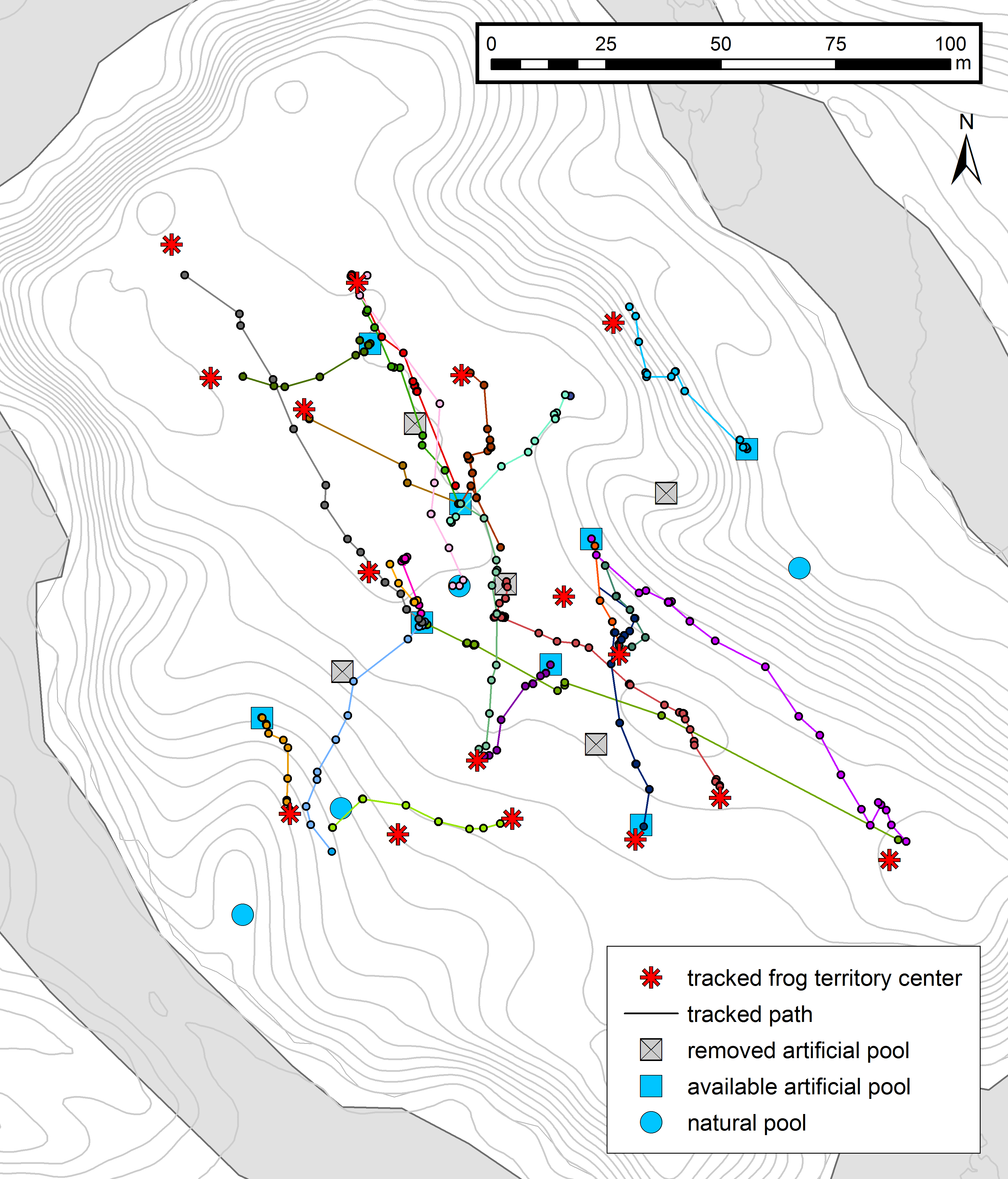

Supplement: Figure S2 — Trajectory map showing movement patterns of frogs homing back to their territory after tadpole transport. Red asterisks represent the territory centers of tracked carriers and colored lines show different tracking events. Squares represent the cross-array of thirteen artificial tadpole deposition sites, blue squares representing available pools and gray crossed squares the removed deposition sites. Blue circles represent four potential natural pools, which were visited by tadpole carriers during tracking. Contour lines (1 m) and the Arataye River are drawn in light gray. [file peerj-05-3745-s003.png]

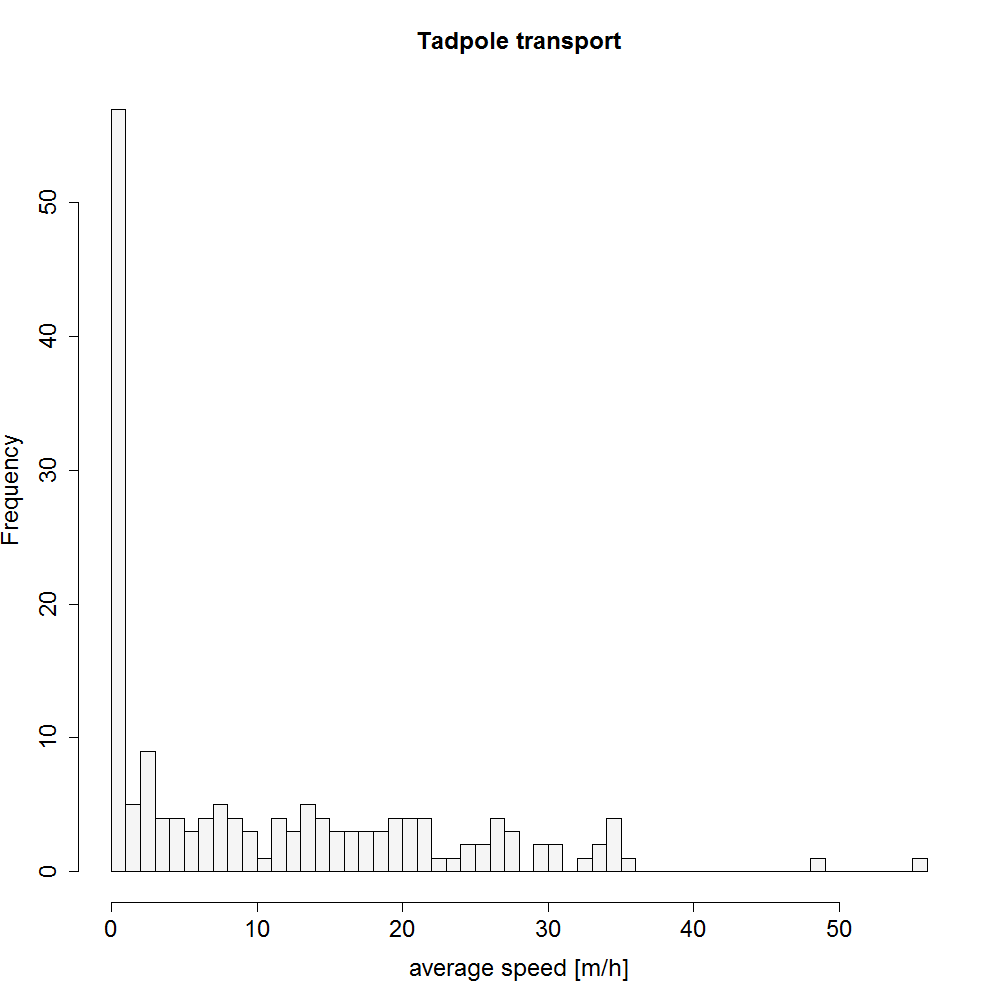

Supplement: Figure S3 — Histogram showing the range of movement speed during tadpole transport from one tracking location to the next one (m/h). [file peerj-05-3745-s004.png]

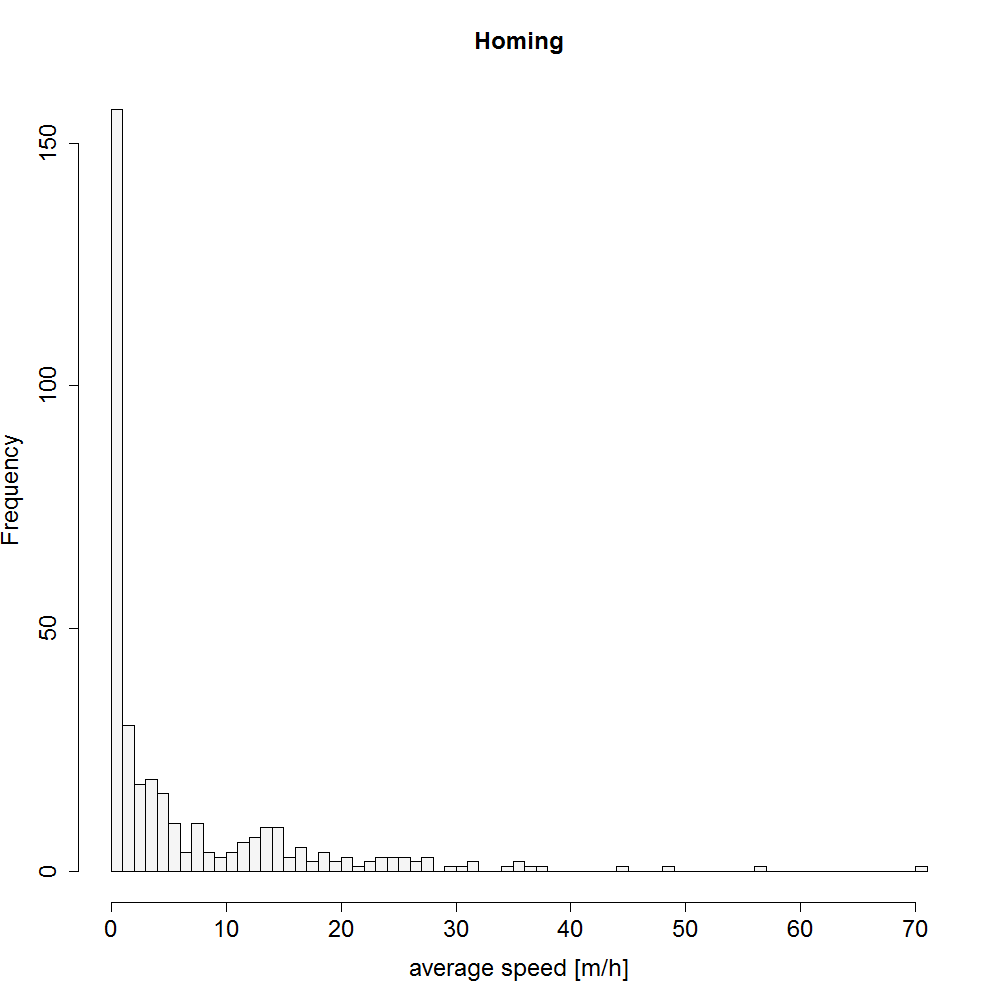

Supplement: Figure S4 — Histogram showing the range of movement speed during homing from one tracking location to the next one (m/h). [file peerj-05-3745-s005.png]
