# Supplementary material for: Relying on known or exploring for new? Movement patterns and reproductive resource use in a tadpole-transporting frog
Source: PeerJ. 2017 Aug 29;5:e3745. doi: 10.7717/peerj.3745 (PMC5580388; doi:10.7717/peerj.3745)
Supplement: Table S1 — File contains Table S1 on model selection, Table S2 showing all model-averaged coefficients and the discussion of the model results. [file peerj-05-3745-s006.docx]

**Table S1**. Model selection of the analyses examining which factors influence the average speed during periods of three hours (individual identity was fitted as random factor). We followed a multimodel inference approach and retained four models within ∆AICc ≤ 2 of the top-ranked model, from which we created model-averaged coefficients (Table S2). Df = degrees of freedom, LogLik = log-likelihood, ∆AICc = difference of the second order Akaike’s Information Criterion between the best model and each other possible model, weight = model weight.

| Explanatory variables of each model | df | logLik | ∆AICc | weight |
| --- | --- | --- | --- | --- |
| Tadpoles present, time of the day, temperature | 8 | -315.58 | 0.00 | 0.42 |
| Tadpoles present, time of the day, temperature, rain | 9 | -315.03 | 1.29 | 0.22 |
| Time of the day, temperature | 7 | -317.50 | 1.48 | 0.20 |
| Tadpoles present, rain | 5 | -319.94 | 1.80 | 0.17 |

**Table S2.** Model-averaged coefficients (full-model averaging) with unconditional standard errors SE and relative importance RI (i.e. sum of Akaike weights for each model in which the variable appears) of the best models to explain how the factors influence the average speed during periods of three hours (Table S1). The intercept represents “tadpoles present (TTS)” and “time of the day 0700-1000”.

| Parameter | Estimate | SE | RI |
| --- | --- | --- | --- |
| Intercept | 5.12 | 2.27 |  |
| Temperature | -0.15 | 0.10 | 0.83 |
| Time 1000-1300 | 0.67 | 0.46 | 0.83 |
| Time 1300-1600 | 0.82 | 0.50 | “ |
| Time 1600-1900 | 0.97 | 0.55 | “ |
| No tadpoles (HS) | 0.38 | 0.29 | 0.80 |
| Rain | 0.09 | 0.17 | 0.39 |

Discussion of the models on frog speed

All explanatory variables from the full model remained in the averaged model and thus influence the speed of frogs, with temperature and time having the highest relative importance, closely followed by tadpole carrying and rain with the least relative importance (Table S2). Frogs moved in general fastest during the late afternoon (between 1600–1900 h). Calling activity is higher during this period of time (Kaefer et al., 2012) which might motivate male frogs to return faster to their territory. The breeding season of many tropical anurans coincides with the rainy season, characterized by higher humidity levels and lower temperatures compared to the dry season (Cynthia, Uetanabaro & Haddad, 2005). During tadpole transport, lower temperatures and a higher amount of rain could both cause less energy expenditures during locomotion (Navas, Gomes & Carvalho, 2008). The effects of tadpole presence are discussed in the main text. Future research, using fine-scale weather stations on the forest floor, will be necessary to investigate in more detail the effects of weather conditions on the movement patterns of poison frogs.

**References**

Cynthia P de A., Uetanabaro M., Haddad CFB. 2005. Breeding activity patterns, reproductive modes, and habitat use by anurans (Amphibia) in a seasonal environment in the Pantanal, Brazil. *Amphibia-Reptilia* 26:211–221

Kaefer IL., Montanarin A., Da Costa RS., Lima AP. 2012. Temporal patterns of reproductive activity and site attachment of the brilliant-thighed frog *Allobates femoralis* from central Amazonia. *journal of Herpetology* 46:549–554. DOI: http://dx.doi.org/10.1670/10-224

Navas CA., Gomes FR., Carvalho JE. 2008. Thermal relationships and exercise physiology in anuran amphibians: integration and evolutionary implications. *Comparative Biochemistry and Physiology Part A: Molecular & Integrative Physiology* 151:344–362
