# Supplementary material for: Relying on known or exploring for new? Movement patterns and reproductive resource use in a tadpole-transporting frog
Source: PeerJ. 2017 Aug 29;5:e3745. doi: 10.7717/peerj.3745 (PMC5580388; doi:10.7717/peerj.3745)
Supplement: Table S3 — Each row represents a specific tracking event. Columns show the frog ID, number of tadpoles, tracked distance, total time of the tracked path (h), average speed (m/h) across the entire TTs, number of deposition sites visited (in parentheses the number of available (a) and non-available (n) deposition sites), total estimated tadpole transport distance, straight-line distance (from the territory centroid across all deposition site until all tadpoles were deposited) and a straightness coefficient. [file peerj-05-3745-s007.docx]

| **Frog**  **ID** | **Tadpoles** | **Tracked distance (m)** | **Duration (h)** | **Speed (m/h)** | **Deposition sites visited** | **Estimated tadpole transport distance (m)** | **Straight line distance (m)** | **SC** |
| --- | --- | --- | --- | --- | --- | --- | --- | --- |
| i13-003 | 2 | 94.1 | 5.42 | 17.36 | 2  (a=1,n=1) | 94.1 | 89.45 | 0.95 |
| i13-003 | 9 | 35.32 | 5.38 | 6.57 | 1 | 35.32 | 32.82 | 0.93 |
| i13-027 | 9 | 24.68 | 2.08 | 11.87 | 1 | 72.95 | 22.78 | 0.92 |
| i14-017 | 6 | 35.06 | 4.83 | 7.26 | 2  (a=1,n=1) | 56.76 | 20.02 | 0.57 |
| i14-038 | 11 | 22.67 | 3.13 | 7.24 | 2  (a=1,n=1) | 55.59 | 20.27 | 0.89 |
| i15-010 | 3 | 126.43 | 8.5 | 14.87 | 2  (n=2) | 126.43 | 99.42 | 0.79 |
| i15-011 | 13 | 20.96 | 3.08 | 6.81 | 2  (a=1,n=1) | 53.60 | 20.18 | 0.96 |
| i15-011 | 15 | 55.16 | 3.08 | 17.91 | 2  (a=2) | 93.13 | 42.78 | 0.78 |
| i15-016 | 8 | 81.44 | 7 | 11.63 | 4  (a=3,n=1) | 166.43 | 69.39 | 0.85 |
| i15-016 | 10 | 47.78 | 7.27 | 6.57 | 4  (a=2,n=2) | 142.94 | 41.06 | 0.86 |
| i15-025 | 14 | 141.37 | 13.25 | 10.67 | 4  (a=2,n=2) | 154.75 | 73.84 | 0.52 |
| i15-025 | 10 | 22.466 | 7.58 | 2.96 | 2  (a=1,n=1) | 55.44 | 20.02 | 0.89 |
| i15-028 | 9 | 59.37 | 4.37 | 13.59 | 3  (a=1,n=2) | 73.85 | 52.64 | 0.89 |
| i15-037 | 10 | 53.87 | 6.43 | 8.38 | 3  (a=2,n=1) | 87.68 | 47.01 | 0.87 |
| i15-037 | 3 | 24.41 | 3.67 | 6.65 | 2  (a=1,n=1) | 46.65 | 20.27 | 0.83 |
| **average** | 9 | 56.34 | 5.67 | 10.16 | 2.4 | 87.71 | 46.57 | 0.83 |
