# Supplementary material for: Relying on known or exploring for new? Movement patterns and reproductive resource use in a tadpole-transporting frog
Source: PeerJ. 2017 Aug 29;5:e3745. doi: 10.7717/peerj.3745 (PMC5580388; doi:10.7717/peerj.3745)
Supplement: Table S4 — Each row represents a specific tracking event. Columns show the tracked distance during homing, straight line distance (from the last deposition site to the territory center), duration of homing, whether homing took more than one day (overnight = 1), average speed and the straightness coefficient. [file peerj-05-3745-s008.docx]

| **Frog**  **ID** | **Distance (m)** | **Straight line**  **(m)** | **Duration**  **(h)** | **Overnight** | **Speed**  **(m/h)** | **SC** |
| --- | --- | --- | --- | --- | --- | --- |
| i13-003 | 22.16 | 21.84 | 1 |  | 22.16 | 0.99 |
| i13-003 | 24.62 | 23.83 | 3.03 |  | 8.13 | 0.97 |
| i13-027 | 26.67 | 24.50 | 6.17 |  | 4.33 | 0.92 |
| i13-027 | 58.03 | 53.33 | 8.25 | 1 | 7.03 | 0.92 |
| i14-017 | 39.48 | 37.80 | 3.17 |  | 12.45 | 0.96 |
| i14-038 | 56.31 | 50.5 | 8.63 | 1 | 6.53 | 0.87 |
| i15-007 | 40.66 | 36.55 | 7.97 |  | 5.10 | 0.9 |
| i15-009 | 101.26 | 97.72 | 8.08 | 1 | 12.53 | 0.97 |
| i15-010 | 83.89 | 63.82 | 49.9 | 1 | 1.68 | 0.76 |
| i15-011 | 23 | 20.68 | 5 |  | 4.6 | 0.9 |
| i15-016 | 109.58 | 94.84 | 19.08 |  | 5.74 | 0.87 |
| i15-016 | 123.46 | 113.82 | 19.5 | 1 | 6.33 | 0.92 |
| i15-020 | 63.38 | 52.52 | 20.6 | 1 | 3.08 | 0.83 |
| i15-024 | 34.92 | 28.57 | 7.12 |  | 4.91 | 0.82 |
| i15-025 | 57.99 | 54.90 | 5.83 |  | 9.95 | 0.95 |
| i15-025 | 57.68 | 50.88 | 9 |  | 6.41 | 0.88 |
| i15-025 | 76.6 | 69.78 | 4.67 |  | 16.40 | 0.91 |
| i15-028 | 72.82 | 28.27 | 22.5 | 1 | 3.24 | 0.39 |
| i15-029 | 51.49 | 40.04 | 11.33 | 1 | 4.55 | 0.78 |
| i15-030 | 43.77 | 37.04 | 6.5 | 1 | 6.73 | 0.85 |
| i15-037 | 16.74 | 14.33 | 5.73 | 1 | 2.92 | 0.86 |
| i15-037 | 15.98 | 14.31 | 4 |  | 4 | 0.9 |
| **average** | 54.57 | 46.81 | 10.78 |  | 7.22 | 0.87 |
