# Supplementary material for: Relying on known or exploring for new? Movement patterns and reproductive resource use in a tadpole-transporting frog
Source: PeerJ. 2017 Aug 29;5:e3745. doi: 10.7717/peerj.3745 (PMC5580388; doi:10.7717/peerj.3745)
Supplement: Table S5 — Summarized data of the average angular deviation, average distance from the straight-line path and SC for the tadpole transport of frogs which encountered only available pools during tadpole transport (A) and frogs which encountered non-available pools (N), minimum, maximum, 1st and 3rd quartile, median and mean. [file peerj-05-3745-s009.docx]

|  | **Pool availability** | **Min.** | **1st Qu.** | **Median** | **Mean** | **3rd Qu.** | **Max.** |
| --- | --- | --- | --- | --- | --- | --- | --- |
| **Angular deviation** | **N** | 2.02 | 4.76 | 13.08 | 34.68 | 34.46 | 136.4 |
|  | **A** | 0 | 5.22 | 18.17 | 24.84 | 36.39 | 98.54 |
| **Distance to straight line** | **N** | 0.15 | 0.32 | 1.93 | 1.82 | 2.53 | 4.18 |
|  | **A** | 0.16 | 0.41 | 0.92 | 2.05 | 3.53 | 6.68 |
| **SC** | **N** | 0.9 | 0.95 | 0.96 | 0.96 | 0.998 | 0.998 |
|  | **A** | 0.06 | 0.88 | 0.94 | 0.87 | 0.99 | 0.999 |
