# Supplementary material for: Relying on known or exploring for new? Movement patterns and reproductive resource use in a tadpole-transporting frog
Source: PeerJ. 2017 Aug 29;5:e3745. doi: 10.7717/peerj.3745 (PMC5580388; doi:10.7717/peerj.3745)
Supplement: Table S6 — Summarized data of the average angular deviation, average distance from the straight-line path and SC for the homing trajectories of frogs which encountered only available pools during tadpole transport (A) and frogs which encountered non-available pools (N), minimum, maximum, 1st and 3rd quartile, median and mean. [file peerj-05-3745-s010.docx]

|  | **Pool availability** | **Min.** | **1st Qu.** | **Median** | **Mean** | **3rd Qu.** | **Max.** |
| --- | --- | --- | --- | --- | --- | --- | --- |
| **Angular deviation** | **N** | 4.32 | 20.77 | 29.39 | 29.22 | 41.74 | 43.95 |
|  | **A** | 4.84 | 14.18 | 25.74 | 27.41 | 36.94 | 63.94 |
| **Distance to straight line** | **N** | 0.04 | 0.94 | 1.35 | 1.53 | 1.8 | 3.45 |
|  | **A** | 0.49 | 0.94 | 1.93 | 1.84 | 2.45 | 4.25 |
| **SC** | **N** | 0.39 | 0.86 | 0.9 | 0.84 | 0.91 | 0.96 |
|  | **A** | 0.78 | 0.84 | 0.91 | 0.89 | 0.94 | 0.99 |
